# Introduction: Healthcare Practitioners’ Emotions and the Politics of Well-Being in Twentieth Century Anglo-America

**DOI:** 10.1093/jhmas/jrad023

**Published:** 2023-05-05

**Authors:** Jacob D Moses, Agnes Arnold-Forster, Samuel V Schotland

**Keywords:** history of emotions, affect, health care work, twentieth century, well-being, detached concern, clinical empathy

## Abstract

From the stress of burnout to the gratification of camaraderie, medicine is suffused with emotions that educators, administrators, and reformers have sought to shape. Yet historians of medicine have only begun to analyze how emotions have structured health care work. This introductory essay frames a special issue on health care practitioners’ emotions in the twentieth-century United Kingdom and United States. We argue that the massive bureaucratic and scientific changes in medicine after the Second World War helped to reshape affective aspects of care. The articles in this issue emphasize the intersubjectivity of feelings in healthcare settings and the mutually constitutive relationship between patients’ and providers’ emotions. Bridging the history of medicine with the history of emotion demonstrates how emotions are instilled rather than innate, social as well as personal, and, above all else, change over time. The articles reckon with the power dynamics of healthcare. They address the policies and practices that institutions, organizations, and governments have implemented to shape, govern, or manage the affective experiences and well-being of healthcare workers. And they point to important new directions in the history of medicine.

Medicine can make people miserable. In 1950, General Practitioner Donald McI. Johnson wrote an article for the *British Medical Journal* describing his experiences of clinical training. He implied that the hospital was a type of “total institution,” and criticized the homogeneity and pressures of medical life and work.^2^ Trainee doctors must, he suggested, “acknowledge that the entire universe has contracted itself to the area within these walls, and that one is expected to make the mental surrender to this cosmos.”^3^ While for some, this all-encompassing experience might prove positive and cultivate a sense of belonging, collegiality, or at the very least a kind of “Blitz spirit,” for others — including himself — it was profoundly distressing: “I became the victim of a wild, tearing claustrophobia.” McI. Johnson did not hold his teachers, or even any individual, responsible for how he felt while at work and about his job, instead he blamed “the system.” The “distinguished gentlemen” in whose steps he followed were as much “victims” as he was. McI. Johnson was not alone in feeling this way. Eighteen years later, hospital chaplain George Day observed the phase of “deep despair” experienced by trainee physicians: “Despair that they will ever get on top of their job; that they will ever give satisfaction to their sometimes thoughtless and exacting chiefs; despair that they cannot afford their patients all the unhurried attention they once hoped to be able to give. They often become exhausted in body, mind, and spirit.”^4^ This quote could equally have been written about healthcare professionals working today. The issues Day identified in 1968 are seemingly timeless and universal. Anxieties about workload, hierarchies, and scheduling — as well as feelings of exhaustion, alienation, and frustration — are as much a part of healthcare labor today as they were in the middle of the twentieth century. Questions around “work-life balance,” excessive temporal commitment, and boundaries between leisure and labor also circulate in healthcare settings today. Indeed, concerns about the emotional health and “well-being” of healthcare professionals have gained increased public attention in both Britain and the United States. Even before the Covid-19 pandemic, professional organizations and health policymakers placed new emphasis on issues such as stress, burnout, and bullying. Recent research has demonstrated elevated levels of depression and suicidal ideation amongst doctors and nurses.^5^

However, the emotions experienced by healthcare professionals, the way those feelings have been articulated, and the causes of both satisfaction and distress, are all dependent on where these people have worked and have all shifted since McI. Johnson first penned his article in 1950. Moreover, the methods that have been thought up, debated, or implemented to resolve these emotional problems have also changed. In other words, healthcare practitioners’ emotions and the politics of well-being have a history, and it is this history that we explore in this special issue. The constituent articles deal with a variety of questions, and use a range of case studies. They investigate multiple different professions, institutions, and time periods. They all, albeit in different ways, tackle the emotional cost of care and the idea — pervasive among healthcare professionals of all sorts — that working in clinical environments can be damaging or deleterious to a person’s mental and emotional health and sense of self.^6^ The articles reckon with the power dynamics of healthcare settings, with institutional hierarchies, and with the intersections among social class, gender, race, sexuality, ethnicity, and the emotions. The essays also address the policies and practices that institutions, organizations, and governments have implemented to shape, govern, or manage the affective experiences and well-being of healthcare workers.

However, the emotions felt by doctors, nurses, and other health professionals were not, and have never been, only negative or harmful. Taken together, the articles attest to the sociality of emotions: collegiality, communities of feeling, and the supportive nature of emotional interactions with friends, patients, and co-workers. One of the key contributions made by this special issue is to demonstrate the intersubjectivity of feelings in healthcare settings and the mutually constitutive relationship between patients’ and providers’ emotions. Ideas about vocation, professionalism, and work as a “labor of love” are particularly powerful in clinical settings and are frequently deployed to articulate the distinctiveness or exceptionalism of healthcare work.^7^ Jobs (however stressful or proximate to death and dying) are not just sources of emotional distress, but also can provide people with joy, satisfaction, and meaning. To quote medical anthropologist and physician wellness advocate, John Henry Pfifferling, in 1983: “Model physicians — healthy, successful, joyful practitioners — must be studied and their ‘immunologic’ factors identified and reinforced over the entire gamut of work and training settings.”^8^

This special issue is not a composite history of different, discrete emotions felt by individual healthcare professionals. Instead, the various articles examine the history of experience, or broad, emotional states, regimes, or communities.^9^ They also address emotional rhetoric, the use of feelings in professional identity formation and articulation, and “neoliberal” regimes of emotional health management.^10^

## Histories of Emotions, Healthcare, and Work

In the last few years, humanities scholars and social scientists studying medicine and healthcare have paid more attention to affect theory and the history of emotions.^11^ Historians are increasingly attuned to the feelings and experiences of patients and they have addressed the emotional intensity of healthcare activism. However, less attention has been paid to the feelings of healthcare practitioners and the efforts on behalf of governments, administrators, managers, and policymakers to manage the emotional landscape of twentieth-century healthcare.^12^ This special issue historicizes these issues by exploring the changing nature of well-being as both experience and concept, and by using the past to critically appraise current policies and practices.

We have chosen to focus on emotions in twentieth-century healthcare work. The papers draw out emotional themes in a century characterized, in some quarters, by its “stoic coolness.”^13^ The papers cover the post-World War II decades to the end of the century, a period of both profound expansion and reorganization of healthcare in the US and UK. Historians are still casting light on the emotional contours of this recent past, revealing a remarkably varied affective terrain.^14^ Collectively, and in conversation with historians of medicine and historians of emotions, these papers analyze the experiences of health care practitioners to revise notions that modern medical practice has been — or aspires to be — evacuated of affect.

While scholars have been calling for emotions to be written into history since at least the mid-twentieth century, the dominant postwar social theories in North America and Europe marginalized emotions to favor scientific rationalism that they thought could repair the devastations wrought by fascism.^15^ The role of science and technology has figured so centrally in these accounts of progress that Jan Plamper has even suggested that histories of emotions will have to be written as histories of science.^16^ These papers show why medicine — at the crucible of science, technology, and care — will also have to be a part of these histories of emotions.

In the context of healthcare, critics of biomedicine have identified and lamented the objectifying, atomizing, and reductionist inclinations of this powerful mode of describing and intervening upon bodies.^17^ Even as historians of medicine, science, and technology have challenged the notion of modernity as either a progressive narrative of improvement or eroding decline, emotions have received little attention from historians who have perhaps too readily accepted the notion that scientific objectivity banished affect to the shadows.^18^ Similarly, and as Barbara Rosenwein has argued, “modernity” has proven “a problematic category in the history of emotions.”^19^ The sharp periodization implied by modernity suggests sudden epistemic and emotional ruptures, or even more problematically, it stands to revive troubling grand narratives of a Western “civilizing process” that putatively placed emotions under progressively greater forms of self-regulation.^20^ Analysts of modernity are skeptical that these narratives can account for change over time, while still recognizing the cultural power visions of modernity have mustered.^21^

To take a prominent example from one of the most perceptive analysts of medical modernity, Susan Sontag in *Illness as Metaphor* proposed that scientific understanding and medical treatment would unburden cancer patients from the stigma and shame heaped on them.^22^ We invoke modernity to connote how notions of medical progress were underwritten by the perceived power to eradicate not only disease but also the harmful cultural meanings associated with illness.

Thus, the study of emotions in the context of highly technical and bureaucratic biomedicine offers an important pathway to revising still-dominant assumptions about the role of emotions in health care systems, which were increasingly suffused with scientific discourse throughout the twentieth century.^23^ The study of emotions in this period emphasizes the way that scientific medicine and management of healthcare work cannot be characterized as a displacement of humanistic sentimentality by scientific modernity, or warmth by coolness. Even the most imperious modernist aspirations were never envisioned to apply to all aspects of care nor were their efforts to evacuate emotionality ever perfect. Attuning our analysis to the persistence of emotion in modern healthcare continues to build on this project of questioning the salience of modernity.^24^

## Detached Concern and Affective Education

The tensions between clinical distance and emotional attachment are a major theme of the post-war care work described in the essays in this special issue. The contributing authors track efforts to contain, control, or otherwise shape the affective lives of medical professionals. Sites of training emerge in this collection as important venues of affective education. The notion that medical healers should maintain a demeanor of emotional calmness has been an enduring component of professional identity formation. In an 1889 valedictory address given at the University of Pennsylvania, William Osler advised medical graduates to display “imperturbability,” which he defined as “coolness and presence of mind under all circumstances.” Patients so valued “calmness amid storm,” he warned, that the physician who failed to embody “immobility, impassiveness, or...*phlegm*” would quickly lose their trust. Osler suggested this was a “bodily endowment,” adding regretfully that “congenital defects” could end the careers of physicians unable to adopt this affective disposition.^25^ Osler’s admonition continues to be quoted regularly by medical writers searching for ways to extoll the role of emotional calmness, even as they apply it to storms quite different from those countenanced by Osler.^26^ In this tradition, maintaining emotional coolness is a way of upholding authority and professional power.^27^

Osler’s embrace of clinical detachment was also a gendered choice. Emotional coolness was coded as White and male. By contrast, as women entered British and American organized medicine in the nineteenth century, one of the central arguments cited for their clinical presence in the profession was women’s unique affective capabilities, uniting both science and sympathy.^28^ However, such arguments were also weaponized and used to suggest that women were emotionally volatile, and thus unsuited to the practice of clinical medicine. As the ideals of laboratory medicine gained authority in the late nineteenth century, the emphasis on physicians’ identity and affect shifted, subsuming emotion beneath the supposed objectivity of experimental evidence.^29^ Eventually, trenchant critiques of biomedicine and its objectifying effects came to a head in the 1970s, which is also when women started to (re)gain access to the profession in greater numbers.^30^ Those in the feminist women’s health movement and many female physicians emphasized medicine’s misogyny and dehumanization.^31^ As the essays discuss, gender played a co-constitutive role in shaping healthcare workers’ vocation, emotional identity, and sense of self.

Social analyses of emotions in healthcare have emphasized how affective temperaments are inculcated within institutions. Pioneering work by medical sociologist Renée Fox noted the historical importance of “detached concern” in mid-twentieth-century medical training and practice. Fox first derived her influential concept from field work on the wards of the Peter Bent Brigham Hospital in Boston. Clinical researchers had to balance their concern for the patients’ humanity with the equanimity (or, perhaps, imperturbability) of the experimenter. Fox argued that such a posture served both scientific functions and afforded psychological protections as physicians undertook administering interventions with uncertain chances of saving the lives of the direly ill.^32^ In other words, detached concern was an emotional posture that was produced with the identity of an physician-scientist. Subsequent work by Fox (with psychiatrist and psychoanalyst Howard Lief) noted how this almost paradoxical stance toward patients, combining the “counterattitudes” of objectivity and empathy, was cultivated in medical education. Lief and Fox proposed a sequence of step-like socialization phases that tracked from the anatomy lab to the clinical wards. Yet these steps did not simply represent increased detachment. Rather, standardized rituals of twentieth-century medical education emphasized both attachment with the patient and detachment with the sick body in ways that initially appeared contradictory to students. Too much detachment could lead to cynicism, dejection, or other forms of emotional paralysis. The ability to balance countervailing commitments across medical school experiences thus became the mark of the successful professional.^33^

Over the ensuing decades, it became evident that detached concern was a historically specific pedagogical and professional stance that would yield in response to social, cultural, and political changes. The growth of biomedical experimentalism in the 1950s had necessitated both epistemic and emotional forms of distancing. By the 1970s, however, Fox observed that some medical students placed much greater emphasis on “feeling with the patient” and accepted the necessity for detachment with greater ambivalence.^34^ Indeed in the twenty-first century, “clinical empathy” is held up by many as a pedagogical goal and moral virtue.^35^ But this emphasis on emotional labor, medical sociologists have argued, connotes historical shifts in the corporate and consumer-minded organization of healthcare.^36^ Rather than normatively judging detachment or attachment, crudely splitting emotions into positive or negative valences, such work invites historical scrutiny about why different emotional dispositions have been prized in different areas of health work. Emotional attachments and detachments are essential features of professional preparation. They represent different modes of motivation and affective protection that are inculcated into care work.

Surveying the articles in this issue makes it immediately apparent that the authors have importantly taken an expansive view of healthcare work. In the twentieth century, British and North American practitioners had to negotiate increasingly complex health systems. Authors track hospital administrators, community nurses, and health-activists in addition to medical students and physicians. This expanded aperture captures a broader array of affective dispositions in the health professions. Belying the transhistorical medical humanism invoked by Osler, this issue demonstrates that such emotions are instilled rather than innate, social as well as personal, and above all else, change over time.

Phil Begley’s article spans the Atlantic to show how the emotional climate of medical institutions became an explicit metric of concern for hospital administrators in the US and UK in the early decades of the twentieth century. Begley considers hospital administrators’ role in creating “happy hospitals” that married compassion with managerial efficiency. Against the stereotype of the hospital bureaucrat as merely a cog in a lifeless machine, Begley uncovers how training programs for administrators emphasized “imagination,” “tact,” and “humanity” in envisioning what constituted good management and a happy hospital. And much like the medical workers they managed, the workers Begley studies underwent administrative “clerkship” and “residency” to become acculturated to a workplace where illness, death, and things unsettling in everyday life were routine features of hospital life. The emotional detachment, it was argued, was even more critical for administrators. “Display of temperament,” one hospital director wrote in the 1952 *Principles of Hospital Administration*, “while unfortunate on the part of any hospital worker, or anyone else for that matter, becomes inexcusable when displayed by the administrator.” The administrator who “suffers from jumpy nerves” would fail to earn the confidence of patients and healthcare employees. The principles of administration proscribed “nervous irritability” as “a privilege that should be reserved exclusively for the patients and their relatives.”^37^ This admonition for administrators to become steely and stoic shows how emotional regulation was regarded as a source of professional virtue. Training and the professional identity of a hospital manager were co-constitutive, Begley argues, and both medical and, with increasing authority throughout the twentieth century, professional managers influenced the emotional structures of the hospital management and notions of healthcare efficiency.

Within the logic of functionalist sociology, detached concern was modeled in medical training because this affective posture afforded considerable protections to the practitioners and permitted them to perform their prescribed duties. It, in other words, worked within the system of the hospital. The hospital is indeed a resonant site of health work and central node of training, including in the affects of professionalism. However, the articles in this collection extend beyond the walls of these institutions into the community. Ruth Beecher’s contribution offers a historically grounded critique for how training for community health practitioners in the UK did not work when these professionals were tasked in the 1980s with recognizing the physical and psychological signs of child sexual abuse. Beecher’s analysis is highly attuned to the emotional complexities for health professionals charged with recognizing and responding to signs of rape, incest, and other crimes against children. These reporters fell short of meeting their mandates, Beecher argues, because the training they received emphasized “objective” measures with little explicit consideration for the emotional difficulty of recognizing what these might portend. The detachment of the training material from the considerable concern these matters predictably provoked left community health workers without the knowledge and emotional preparation. An alternative history, Beecher hints, could be found in anti-rape feminist activism, a movement that had drawn attention to the problem of child abuse in the first place.

The association of emotions with feminism — among historical actors and as a mode of writing history — points to the gendered nature of particular emotions and the study of affect writ large. Kim Adams’s contribution to this issue complicates this common association. Adams juxtaposes two influential texts from the 1970s, written in close geographical proximity in very different genres. The blistering satire of *House of God* exposed the crushing effects of residency on medical trainees, while *Our Bodies Ourselves*, a major output of the women’s health movement, revealed the intricacies of patient consciousness. By putting these texts together, Adams not only critiques the portrayal of female patients in the still-widely-read *House of God*, a core text of medicine’s hidden curriculum that offers a kind of affective education, but also reveals surprising commonalities between the works’ invocation of “loose expertise.” Adams argues that both *Our Bodies Ourselves* and *House of God* initiated moves away from traditional medical authority and sought to resist, in very different ways, the perceived dehumanizing aspects of medical institutions.

While Adams uses *Our Bodies Ourselves* to critique how women are portrayed in *House of God*, she notes the longevity of the latter “often positions the sentiments of the interns as a transhistorical given, even as the demographics of medical trainees and the conditions of internships change.” As recently as 2017, ex-doctor and British medical writer Adam Kay called *House of God* “Darkly funny, with dozens of unforgettable moments, it’s the gold-standard of its genre, and should be required reading for anyone about to slap on a stethoscope and step onto the ward for the first time.”^38^ By attending to the affective constructions presented in both narratives — one thinly veiled memoir, the other a health handbook — Adams recovers the affective role of both masculinist and feminist portrayals of medical practice. She also encapsulates another key theme of the special issue, the notion that emotions have the capacity to re-inscribe lines of power within healthcare institutions — whether that is between patient and provider, or between different healthcare professions or kinds of care work. The role that someone occupies within the health system, and how it is valued, depends on the type of emotional style they adopt, the kind of emotional skills they are thought to have, and how they balance their feelings against clinical or technical expertise.

Megann Licskai’s contribution centers on the affective lives of pro-life activists in the US. Licskai argues that an underexamined strand of anti-abortion rhetoric is the pro-life “conversion” narrative, in which former abortion providers and patients retrospectively portray their prior involvement with pregnancy termination as “cold, clinical, and detached” in contrast to the Christian faith at the center of much anti-abortion activism in the US. This rendering attempts to draw a dichotomy between dispassionate science and affective engagement of pro-life communities. For instance, former abortion providers often describe in popular media a process of “unlearning” a mode of clinical detachment that coincided with both religious and medical conversion. However, Licksai deploys evidence from memoirs, speeches, and public writings to challenge this stark distinction, arguing that “there is no easy dichotomy between affect and expertise.” Instead, Lickai argues, affect and expertise in abortion politics have been mutually constitutive. Even efforts to advance putatively objective fields of anti-abortion medicine in “fetology” and definitions of “post-abortion syndrome,” Lickai argues, have been imbued with strategies for eliciting affective responses from the intended medical and lay audiences. In contrast to the anger and violence displayed by some anti-abortion activists, pro-life training manuals in the 1980s and 1990s equipped counsellors with strategies for summoning feelings of guilt, regret, and shame and to provide a channel of achieving emotional catharsis. “Through their emotional responses to abortion,” Licksai writes, “the post-abortion counselor and the post-abortion patient built a medical condition and practice that legitimized the marriage between emotion and science in the pro-life movement.” While this article follows affective strands in the history of anti-abortion activism, it points to important corollaries for supports of abortion and reproductive rights concerning the perceived detachment of medical science in one of the most highly affectively charged domains of US politics.

The special issue closes with a commentary by Scott Podolsky, who offers his perspective as a primary care physician and historian of medicine. Drawing on examples from his own scholarship and practice, he reflects on how emotions ubiquitous in the daily work of health care have been largely unexamined in the historiography of medicine. He notes, “emotions – whether welcome or acknowledged or not – have long shaped clinical encounters and clinical administrative activities alike.”

What do these articles, taken as a collection, tell us about how to historically situate the emotions of care providers? To historians of medicine, disciplinarily attuned to the social structure of healing and professionally wary of the domains of intra-personal thought inaccessible to archival scrutiny, the project of analyzing the affective experiences of healers may appear fraught. Is the study of emotions a retreat to the personal, biographical, or speculative? Quite the contrary. Taken together, the articles in this collection form an argument for how the emotional experience of individual healers are socially configured and historically reconfigured over time. The recurring focus across the papers on training programs — for hospital administrators, community nurses, medical residents — point to the importance of formal and tacit training in inculcating an affective education in power-laden relationships.^39^ Studying the ways that affect is taught and trained has much to teach us as historians of medicine.

## The Covid-19 Pandemic

This special issue was first conceived of at the end of 2019. We were not to know that we would end up writing and editing a collection of essays on the well-being of healthcare practitioners amidst one of the worst crises ever faced by healthcare systems, workers, governments, and citizens. It is, therefore, impossible to introduce this special issue without commenting on the impact of the coronavirus pandemic on healthcare practitioners’ emotional health or on the working lives of academics. As editors, we have been repeatedly impressed by our authors’ ability to develop and deliver such insightful scholarship under extraordinary circumstances. It seems pertinent to acknowledge the extra labor (emotional and otherwise) that working under shelter-in-place orders and lockdowns has demanded. Caring responsibilities, the blurring of boundaries between personal and professional life, and questions about occupational health and well-being are even more pertinent now than they were when we first solicited articles for this collection.

The pandemic has also made these questions more pressing for healthcare professionals and policymakers. While none of the articles address Covid-19 explicitly, they all have direct and indirect bearing on how healthcare practitioners cope with, and feel about their work, particularly during periods of intense stress. The pandemic may well prove to be a turning point in the way people think about and legislate healthcare practitioners’ emotional health, but as the various articles in this special issue demonstrate, the emotional costs of care and the deleterious impact healthcare work can have on people’s well-being is not new.^40^ As Begley’s article demonstrates, since the NHS’s inception, hospitals have operated as incubators for new ideas about organizations’ roles and responsibilities towards the morale or happiness of their staff. In addition, Beecher’s essay make clear a lesson we should all have already learnt from the coronavirus pandemic: that care work can prove emotionally troubling even away from the pressures of hospital life, and that we must retain a focus on non-clinical members of the healthcare workforce. Moreover, ignoring the emotional health of these members of the healthcare ecosystem affects the quality of care that can be provided to patients, influencing their well-being in turn.

Covid-19 has exacerbated and exposed existing social and health inequalities and emphasized a point that many people have known for a long time: that healthcare services and systems are themselves agents of discrimination and abuse. In both Britain and the US, people of color died and continue to die from Covid-19 at much higher rates than their White counterparts, and this was true for healthcare professionals as well. In the NHS, Black and South Asian doctors and nurses were much more likely to get sick and much less likely to survive. In the US, Susan Moore, a Black internist whose medical concerns were infamously dismissed, died of Covid-19.^41^ 95% of British doctors who died were Black or of South Asian origin. Covid-19 did not, of course, create this situation. And as Adams’s article reveals, sexism, racism, and homophobia are embedded in both British and American healthcare systems. Indeed, neither country has adequately reckoned with the institutional racism of their respective services.^42^

Many of the problems built into healthcare systems that cause healthcare workers’ emotional distress well predate Covid-19, including poor working conditions, excessive hours, lack of professional autonomy, embedded hierarchies, and problematic power imbalances. The pandemic presents us with an opportunity to re-think what a caring healthcare system looks like, and to reconfigure the contours of healthcare labor to better serve practitioners for the eventual benefit of patients. However, we must not disregard the past as a potential source of solution. The problem healthcare practitioners have faced this past year are systemic and require systemic remedies, not least a re-imagining of training, education, and selection.

## Further Research

The articles all offer innovative and substantive contributions, and we hope that they will prompt future conversations about, and research into, the intersections between the history of healthcare and the emotions. As a collection of five essays, the special issue could never offer a comprehensive account of the field or address all of the questions we want answered. All of the articles deal with allopathic medicine, and we eagerly anticipate alternative histories of clinical feeling in alternative medicine.^43^ One of the key critiques of allopathic medicine is the kind of unemotional or detached care patients can receive and one of the many attractions of alternative healthcare professionals is their alternative emotional sensibility or methods of interaction with those seeking their advice or treatment. We hope that someone is already researching the feelings of naturopaths or acupuncturists and investigating the emotional costs of providing care in other healing traditions such as Traditional Chinese Medicine or Ayurveda.

By maintaining an Anglo-American focus, we planned to illustrate the similarities, differences, and interconnected nodes between these two nations. The various contributions address issues such as national politics and practitioners’ well-being, the emotional landscape of the NHS versus the US insurance-based systems, and the way affect shapes healthcare cultures and environments. After WWII, the healthcare systems of Britain and the US diverged dramatically and are now structured, administered, and financed very differently. There are, therefore, other healthcare systems that are more similar to each other. And yet, we maintain that there is value in the juxtaposition we have chosen. The two healthcare systems occupy prominent roles in the other nation’s collective imagination. The US’s insurance-based system is frequently held up as a worrying potential alternative to British defenders of their NHS. In contrast, Britain’s socialized healthcare system is either a utopian ideal or an anxiety-producing threat, depending on the American you ask.^44^

However, an expanded geographical scope is much needed. Comparisons between other nations in the Global North would prove fruitful. For example, how does the NHS compare with other free at the point of access healthcare systems in Europe, Australia, New Zealand, or Canada? Similarly, more work on the Global South needs to be done, not just to explore the affective dynamics of the other healing traditions that have their origins there, but also to investigate how allopathic medicine might differ in different national and regional contexts.^45^ Finally, while our articles have looked at non-clinical or nursing members of the healthcare workforce, we think that care work and social care require further study.^46^ This work is especially needed now that Covid-19 has emphasized both our reliance on these systems, and our failings to properly administrate, fund, or value these essential services and their workers.

## Footnotes

2 While McI. Johnson was not referring to the “total institution” in the Goffmanian sense, the concept would later gain intellectual traction. See Erving Goffman, *Asylums: Essays on the Social Situation of Mental Patients and Other Inmates* (New York: Anchor Press, 1961).

3 Donald McI. Johnson, “A G.P. On His Clinical Training,” *British Medical Journal* 2 (1950): 493-496, 494.

4 George Day, “Personal View,” *British Medical Journal* 3 (1968): 182.

5 Rupinder K. Legha, “A History of Physician Suicide in America,” *Journal of Medical Humanities* 33 (2012): 219–244; Dennis Campbell, “NHS Staff Quitting Due to Burnout and Bullying, Report Says,” *Guardian*, 3 June 2019; D. A. Campbell Jr, S. S. Sonnad, F. E. Eckhauser, K. K. Campbell, L. J. Greenfield, “Burnout Among American Surgeons,” *Surgery* 130 (2001): 696-701; Sydney McQueen, Melanie Hammond Mobilio, and Carol-Anne Moulton, “Fractured in Surgery: Understanding Stress as a Holistic and Subjective Surgeon Experience,” *American Journal of Surgery* 221 (2020): 793-798; Abi Rimmer, “Nearly Two Thirds of Doctors have Anxiety or Depression, BMA Survey Finds,” *British Medical Journal* 372 (2021): n22; L. S. Rotenstein, M. Torre, M. A. Ramos, R. C. Rosales, C. Guille, S. Sen, D. A. Mata, “Prevalence of Burnout Among Physicians: A Systematic Review,” *JAMA* 320 (2018): 1131-1150; T. D. Shanafelt, S. Boone, L. Tan, L. N. Dyrbye, W. Sotile, D. Satele, CP West, J. Sloan, M. R. Oreskovich, “Burnout and Satisfaction With Work-Life Balance Among US Physicians Relative to the General US Population,” *Archives of Internal Medicine* 172 (2012):1377–1385.

6 David Nicholas Barron and Elizabeth West, “The Emotional Costs of Caring Incurred by Men and Women in the British Labour Market,” *Social Science and Medicine* 65 (2007): 2160-2171.

7 Historian Michael Brown has looked at the place of emotions in the development of surgeons’ professional identities in the nineteenth-century. See Michael Brown, “Surgery and Emotion: The Era Before Anaesthesia,” *The Palgrave Handbook of the History of Surgery,* ed. Thomas Schlich (London: Palgrave Macmillan, 2017): 327-348; Michael Brown, “Redeeming Mr. Sawbone: Compassion and Care in the Cultures of Nineteenth-Century Surgery,” *Journal of Compassionate Health Care* 4: 13 (2017); Jane Dawson, “A History of Vocation: Tracing a Keyword of Work, Meaning, and Moral Purpose,” *Adult Education Quarterly* 55 (2005): 220-231.

8 John Henry Pfifferling, “The Promotion of Physician Wellbeing,” *North Carolina Medical Journal* 44 (1983): 538-539, 539.

9 The concept “emotional regime” means an emotional community that sanctions certain emotional expressions and experiences whilst censuring others. William Reddy, *The Navigation of Feeling: A Framework for the History of Emotions* (Cambridge: Cambridge University Press, 2001). Reddy, in a similar vein to Arlie Hochschild, suggests that the lack of “emotional liberty” under such regimes causes “emotional suffering” as a result of the suppression of individuals’ true feelings. Arlie Russell Hochschild, *The Managed Heart: Commercialization of Human Feeling* (Berkeley: University of California Press, 2012).

10 Wendy Brown, *Undoing the Demos: Neoliberalism’s Stealth Revolution* (New York: Zone Books, 2015); Ben Anderson, “Neoliberal Affects,” *Progress in Human Geography* 40 (2015): 734–753.

11 Fay Bound Alberti, ed., *Medicine, Emotion and Disease, 1700-1950* (London: Palgrave Macmillan, 2006); Rob Boddice and Bettina Hitzer, ed., *Feeling Dis-ease in Modern History: Experiencing Medicine and Illness* (London: Bloomsbury, 2022); Agnes Arnold-Forster and Alison Moulds, ed., *Feelings and Work in Modern History: Emotional Labour and Labour about Emotions* (London: Bloomsbury, 2022); Agnes Arnold-Forster, Michael Brown, Alison Moulds, “Health, Policy and Emotion,” *BMJ Medical Humanities* 48 (2022): 389-393. See also Thomas Dixon’s Wellcome Trust Collaborative award, “Living with Feeling,” Queen Mary, University of London, https://projects.history.qmul.ac.uk/livingwithfeeling/.

12 Historians have attended to the emotions of nineteenth-century surgery and medicine, for example, Dr Michael Brown’s Wellcome Trust Investigator Award, “Surgery & Emotion,” University of Roehampton, https://www.surgeryandemotion.com. In the context of the twentieth century, see Agnes Arnold-Forster, “The Social and Emotional World of Twentieth Century Anglo-American Surgery: The James IV Association,” *Bulletin of the History of Medicine* 96 (2022): 71-101.

13 William Reddy, “Historical Research on the Self and Emotions,” *Emotion Review* 1 (2009): 302-315.

14 For example, 1950 was set as the terminus date for Bound Alberti, *Medicine, Emotion and Disease*.

15 In social history, see Lucien Febvre’s 1941 essay, “Sensibility and History: How to Reconstitute the Emotional Life of the Past,” in *A New Kind of History: Writings of Febvre*, ed. Peter Burke, trans. K. Folca (New York: Torchbooks, 1973), 12-26. In history of medicine, George Rosen, “People, Disease, and Emotion: Some Newer Problems for Research in Medical History,” *Bulletin of the History of Medicine* 41 (1967): 5–23. On the response to fascism, see Frank Biess and Daniel M. Gross, ed., *Science and Emotions After 1945: A Transatlantic Perspective* (Chicago: University of Chicago Press, 2014), 5.

16 Jan Plamper, quoted in idem., 11.

17 On biomedicalization, see Adele E. Clarke, Laura Mamo, Jennifer Ruth Fosket, Jennifer R. Fishman, Janet K. Shim, ed., *Biomedicalization: Technoscience, Health, and Illness in the US* (Durham: Duke University Press, 2010).

18 Paul White, “Introduction: The Emotional Economy of Science,” *Isis* 100 (2009): 792–797. One of the most comprehensive challenges to this notion is advanced in Lorraine Daston and Peter Galison, *Objectivity* (New York: Zone Books, 2007).

19 Barbara H. Rosenwein, “Modernity: A Problematic Category in the History of Emotions,” *History and Theory* 53 (2014): 69–78. See also Barbara H. Rosenwein, “Worrying About Emotions in History,” *American Historical Review* 107 (2002): 821–845; Fay Bound Alberti, “Emotions in the Early Modern Medical Tradition,” in *Medicine, Emotion and Disease*; Eugenia Lean’s comments in Nicole Eustace et al., “AHR Conversation: The Historical Study of Emotions,” *American Historical Review* 117 (2012): 1487–1531.

20 Jan Plamper, *The History of Emotions: An Introduction* (Oxford: Oxford University Press, 2015); Norbert Elias, trans. Edmund Jephcott, *The Civilizing Process* (New York: Pantheon Books, 1982).

21 Sheila Jasanoff and Sang-Hyun Kim, ed., *Dreamscapes of Modernity: Sociotechnical Imaginaries and the Fabrication of Power* (Chicago: University of Chicago Press, 2015).

22 Susan Sontag, *Illness as Metaphor* (New York: Farrar, Straus and Giroux, 1978).

23 Agnes Arnold-Forster, “‘A Small Cemetery’: Death and Dying in the Contemporary British Operating Theatre,” *Medical Humanities* 46 (2020): 278–287; Janet Golden and Emily K. Abel, “Modern Medical Science and the Divine Providence of God: Rethinking the Place of Religion in Postwar U.S. History,” *Journal of the History of Medicine and Allied Sciences* 69 (2014): 580-603.

24 Bruno Latour, *We Have Never Been Modern*, trans. Catherine Porter (Cambridge, MA: Harvard University Press, 1993).

25 William Osler, *Aequanimitas: With Other Addresses to Medical Students, Nurses and Practitioners of Medicine* (Philadelphia: P. Blakiston, 1922), 4.

26 Matthew L. Edwards, “On William Osler, Psychiatry, and the Law: Reflections on ‘Aequanimitas’ at 130 Years,” *American Journal of Psychiatry Residents’ Journal* 15 (2019): 3; Daniel K. Sokol, “How to Be a Cool Headed Clinician,” *BMJ* 344 (2012): e3980; David L. Anders, “Aequanimitas Centennial,” *Southern Medical Journal* 82 (1989): 1403–1404. Edwards and Sokol both reference “Aequanimitas” in the context of medical malpractice proceedings. On the cultural history of “coolness” in the US, see Peter N. Stearns, *American Cool: Constructing a Twentieth-Century Emotional Style* (New York: New York University Press, 1994).

27 By contrast, see Courtney E. Thompson, “Beyond Imperturbability: The Nineteenth-Century Medical Casebook as Affective Genre,” *Bulletin of the History of Medicine* 96 (2022): 182-210.

28 On sympathy, see Regina Markell Morantz-Sanchez, *Sympathy and Science*: *Women Physicians in American Medicine* (Oxford: Oxford University Press, 1985).

29 John Harley Warner, *The Therapeutic Perspective: Medical Practice, Knowledge, and Identity in America, 1820-1885* (Cambridge, MA: Harvard University Press, 1986). On antimodern discourse in medicine, see John Harley Warner, “The Aesthetic Grounding of Modern Medicine,” *Bulletin of the History of Medicine* 88 (2014): 1-47.

30 See Ellen S. More, Elizabeth Fee, and Manon Parry, ed., *Women Physicians and the Cultures of Medicine* (Baltimore: Johns Hopkins University Press, 2008).

31 On feminist health movement, see Jennifer Nelson, *More than Medicine: A History of the Feminist Women’s Movement* (New York: New York University Press, 2015). On humanization-dehumanization discourse, see Victoria Bates, “Yesterday’s Doctors: The Human Aspects of Medical Education in Britain,” *Medical History* 61 (2017): 48-65.

32 Renée C. Fox, *Experiment Perilous: Physicians and Patients Facing the Unknown* (Glencoe, Ill: Free Press, 1959).

33 Harold I. Lief and Renée C. Fox, “Training for ‘Detached Concern’ in Medical Students,” in *The Psychological Basis of Medical Practice*, ed. Harold I. Lief, Victor F. Lief, and Nina R. Lief (New York: Harper & Row, 1963), 12–35. On “affective neutrality” in medical education, see Allen C. Smith and Sherryl Kleinman, “Managing Emotions in Medical School: Students’ Contacts with the Living and the Dead,” *Social Psychology Quarterly* 52 (1989): 56–69; Mary-Jo DelVecchio Good and Byron J. Good, “Disabling Practitioners: Hazards of Learning to Be a Doctor in American Medical Education,” *American Journal of Orthopsychiatry* 59 (1989): 303–309. Charles S. Bryan has argued that Osler’s call for coolness was not at the expense of empathy, “‘Aequanimitas’ Redux: William Osler on Detached Concern versus Humanistic Empathy,” *Perspectives in Biology and Medicine* 49 (2006): 384–392.

34 Renée C. Fox, “Is There a ‘New’ Medical Student? A Comparative View of Medical Socialization in the 1950s and the 1970s,” in *Essays in Medical Sociology: Journeys into the Field* (New York: John Wiley & Sons, 1979), 100.

35 Jodi Halpern, *From Detached Concern to Empathy: Humanizing Medical Practice* (Oxford: Oxford University Press, 2001).

36 Alexandra H. Vinson and Kelly Underman, “Clinical Empathy as Emotional Labor in Medical Work,” *Social Science and Medicine* 251 (2020): 112904; Carter Hardy, “Clinical Sympathy: The Important Role of Affectivity in Clinical Practice,” *Medicine, Health Care and Philosophy* 22 (2019): 499–513; Kelly Underman and Laura E. Hirshfield, “Detached Concern? Emotional Socialization in Twenty-First Century Medical Education,” *Social Science and Medicine* 160 (2016): 94–101.

37 McGibbony, 184-187, quoted in Begley article.

38 “Doctor’s Orders: Adam Kay Prescribes the Best Medical Reads,” *Waterstones*, 21 September 2017, https://www.waterstones.com/blog/adam-kay-introduces-this-is-going-to-hurt-and-prescribes-the-best-medical-reads.

39 Kelly Underman, *Feeling Medicine: How the Pelvic Exam Shapes Medical Training* (New York: New York University Press, 2020); Rachel Prentice, *Bodies in Formation: Remaking Anatomy and Surgery Education* (Durham, NC: Duke University Press, 2013); Kenneth M. Ludmerer, *Learning to Heal: The Development of American Medical Education* (New York: Basic Books, 1985).

40 Agnes Arnold-Forster, Jacob D. Moses, Samuel V. Schotland, “Obstacles to Physicians’ Emotional Health: Lessons from History,” *New England Journal of Medicine* 386 (2022): 4-7.

41 John Eligon, “Black Doctor Dies of Covid-19 After Complaining of Racist Treatment,” *New York Times*, 23 December 2020, https://www.nytimes.com/2020/12/23/us/susan-moore-black-doctor-indiana.html.

42 An example of the entrenched racism in organized medicine is the backlash to a 2021 JAMA podcast, in which the journal’s deputy editor dismissed structural racism as “an unfortunate term.” For more on the scandal and the AAHM’s official response, see “AAHM calls for properly archiving JAMA’s podcast on ‘’Structural Racism for Doctors,” American Association for the History of Medicine, 30 March 2021, https://www.histmed.org/announcements/news/aahm-news-aahm-calls-for-properly-archiving-jamas-podcast-on-structural-racism-for-doctors-3226.

43 Our thanks to Eana Meng, whose language we have borrowed.

44 Caitjan Gainty, “Why Medicare-for-all Isn’t Enough,” *Washington Post*, 19 August 2019, https://www.washingtonpost.com/outlook/2019/08/19/why-universal-health-care-isnt-enough/.

45 Nicole Elizabeth Barnes, *Intimate Communities: Wartime Healthcare and the Birth of Modern China, 1937-1945* (Berkeley: University of California Press, 2018); Steven Server, “A Test of Conscience: Navigating Mexico’s Servico Médico-Social (1935-1940)” (PhD diss., University of Chicago, 2022).

46 Jennifer Klein and Eileen Boris, *Caring for America: Home Health Workers in the Shadow of the Welfare State* (Oxford: Oxford University Press, 2015); Gabriel Winant, *The Next Shift: The Fall of Industry and the Rise of Health Care and Rust Belt America* (Cambridge, MA: Harvard University Press, 2021).

